# Protein tyrosine phosphatase PTPN22 regulates LFA-1 dependent Th1 responses

**DOI:** 10.1016/j.jaut.2018.07.008

**Published:** 2018-11

**Authors:** Cristina Sanchez-Blanco, Fiona Clarke, Georgina H. Cornish, David Depoil, Stephen J. Thompson, Xuezhi Dai, David J. Rawlings, Michael L. Dustin, Rose Zamoyska, Andrew P. Cope, Harriet A. Purvis

**Affiliations:** a Centre for Inflammation Biology and Cancer Immunology, Faculty of Life Sciences and Medicine, King's College London, London, United Kingdom; b Kennedy Institute of Rheumatology, Nuffield Department of Orthopaedics, Rheumatology and Musculoskeletal Sciences, University of Oxford, Oxford, United Kingdom; c Seattle Children's Research Institute, Departments of Pediatrics and Immunology, University of Washington School of Medicine, Seattle, WA, USA; d Institute of Immunology and Infection Research, Centre for Immunity, Infection and Evolution, University of Edinburgh, Edinburgh, United Kingdom

**Keywords:** PTPN22, IFNγ, LFA-1, T-cell activation, Dendritic cells, Autoimmunity, BMDC, bone marrow derived dendritic cell, DC, dendritic cell, ICAM-1, intracellular adhesion molecule 1, LFA-1, lymphocyte function-associated antigen 1, LN, lymph node, PTPN22, protein tyrosine phosphatase non-receptor -22, RA, rheumatoid arthritis, s.e.m, standard error of mean, s.d, standard deviation, TLR, toll-like receptor, WT, wild-type

## Abstract

A missense C1858T single nucleotide polymorphism within *PTPN22* is a strong genetic risk factor for the development of multiple autoimmune diseases. *PTPN22* encodes a protein tyrosine phosphatase that negatively regulates immuno-receptor proximal Src and Syk family kinases. Notably, PTPN22 negatively regulates kinases downstream of T-cell receptor (TCR) and LFA-1, thereby setting thresholds for T-cell activation. Alterations to the quality of TCR and LFA-1 engagement at the immune synapse and the regulation of downstream signals can have profound effects on the type of effector T-cell response induced. Here we describe how IFNγ^+^ Th1 responses are potentiated in *Ptpn22*^*−/−*^ T-cells and in T-cells from mice expressing *Ptpn22*^*R619W*^ (the mouse orthologue of the human genetic variant) as they age, or following repeated immune challenge, and explore the mechanisms contributing to the expansion of Th1 cells. Specifically, we uncover two LFA-1-ICAM dependent mechanisms; one T-cell intrinsic, and one T-cell extrinsic. Firstly, we found that *in vitro* anti-CD3/LFA-1 induced Th1 responses were enhanced in *Ptpn22*^*−/−*^ T-cells compared to WT, whereas anti-CD3/anti-CD28 induced IFNy responses were similar. These data were associated with an enhanced ability of *Ptpn22*^*−/−*^ T-cells to engage ICAM-1 at the immune synapse when incubated on planar lipid bilayers, and to form conjugates with dendritic cells. Secondly, we observed a T-cell extrinsic mechanism whereby repeated stimulation of WT OT-II T-cells with LPS and OVA_323-339_ pulsed *Ptpn22*^*−/−*^ bone marrow derived dendritic cells (BMDCs) was sufficient to enhance Th1 cell development compared to WT BMDCs. Furthermore, this response could be reversed by LFA-1 blockade. Our data point to two related but distinct mechanisms by which PTPN22 regulates LFA-1 dependent signals to enhance Th1 development, highlighting how perturbations to PTPN22 function over time to regulate the balance of the immune response.

## Highlights

• *PTPN22*^*R620W*^ is one of the strongest risk factors for multiple autoimmune diseases.
• In *Ptpn22*^*−/−*^ and *Ptpn22*^*R619W*^ mice IFNy+ Th1 cells preferentially and significantly expand with age or following immune challenge.
• PTPN22 negatively regulates IFNγ^+^ Th1 cells by T-cell and dendritic cell LFA-1-ICAM-1 dependent mechanisms.
• PTPN22 negatively regulates LFA-1 induced Th1 cells enhancing T-cell LFA-1 clustering and immune synapse formation.
• Repeated stimulation of T-cells with *Ptpn22*^*−/−*^ BMDC enhances Th1 responses.

## 1 Introduction

The non-synonymous *PTPN22* polymorphism C1858T (encoding R620W) is a strong risk factor for the development of multiple autoimmune diseases, including rheumatoid arthritis (RA), type I diabetes, systemic lupus erythematosus, and juvenile idiopathic arthritis [1]. *PTPN22* encodes a tyrosine phosphatase that negatively regulates Src and Syk family kinase (SFK) activity downstream of immuno-receptor signalling cascades [2]. It has become apparent that PTPN22 regulates many pathways in different cell types including the T-cell receptor [3], B-cell receptor [4], integrins [5], as well as dectin-1 [6] and Toll-Like Receptor (TLR) signalling pathways [[7], [8], [9], [10]]. While it has become widely accepted that the autoimmune associated *PTPN22*^R620W^ variant displays reduced binding to the negative regulatory tyrosine kinase Csk due to a missense mutation in the P1 domain [2,11], our understanding as to precisely how the R620W variant affects PTPN22 function remains incomplete. Gain- and loss-of-phosphatase function effects have been reported for the R620W variant, depending on the cellular context and signalling pathway being investigated [5,[10], [11], [12]].

In the peripheral secondary lymphoid organs, *Ptpn22*^−/−^ mice exhibit homeostatic expansion of CD4^+^ and CD8^+^ effector T-cells [3]. Expansion occurs post-thymic selection in an age dependent manner, and is considered to be caused primarily by the TCR being hyper-responsive to antigen stimulation [1]. Furthermore, PTPN22 has a greater impact on the regulation of effector/memory T-cell responses, rather than naïve T-cells, and this is most evident when *Ptpn22*^*−/−*^ T-cells are engaged by MHC molecules presenting lower affinity peptide antigens or low avidity anti-CD3/anti-CD28 stimulation, resulting in enhanced T-cell Ca^2+^ flux and proliferation [13,14]. In addition to regulating T-cell proliferation, the quality of TCR signalling also determines effector T-cell responses, and perturbations to these pathways are capable of exerting profound effects on the type of immune response initiated [15]. Indeed, multiple studies have observed that, by modulating TCR signalling thresholds, PTPN22 negatively regulates the expansion of peripheral regulatory T-cells [14], and is also capable of modulating Th17 to Th1/Treg switching [16]. Therefore, alterations to PTPN22, as conferred by *PTPN22*^*R620W*^ may impact both the quantity and quality of T-cell immune responses, thereby conferring increased risk of autoimmunity.

Previous investigations have demonstrated that PTPN22 is dispensable for Th1 generation *in vitro* in response to CD3 and CD28 stimulation [14]. However, in addition to CD3 and CD28, the integrin LFA-1 also participates in immune synapse stabilisation, and engagement of LFA-1 via ICAM-1 contributes to costimulatory signals transduced in T cells [17]. Our recent investigations have revealed that PTPN22 negatively regulates LFA-1 signalling and T-cell adhesion [5]. Furthermore, multiple studies have demonstrated that LFA-1 engagement is a potent inducer of IFNγ^+^ expression during Th1 cell induction [18,19]. Here, we present data indicating that PTPN22 operates in both a T-cell intrinsic and extrinsic manner to negatively regulate LFA-1 dependent induction of Th1 cells.

## 2 Methods

### 2.1 Mice

Wild type (WT) C57BL/6, *Ptpn22*^−/−^, *Ptpn22*^R619W^ and OT-II TCR transgenic mice were maintained under specific pathogen free (SPF) conditions and used in experiments according to UK Home Office approved protocols. *Ptpn22*^−/−^ mice and *Ptpn22*^R619W^ mutant mice were backcrossed for more than 10 generations to the C57BL/6 strain. Their generation and genotyping have been previously described in detail [4,20]. Age and gender-matched mice were used at either 2–4 month or >12 months in all experiments, as indicated.

### 2.2 Induction and assessment of collagen induced arthritis

The induction of collagen induced arthritis (CIA) in the C57BL/6 strain was performed as previously published [17]. Male WT and *Ptpn22*^*−/−*^ mice of 10–14 weeks of age were injected intradermally at the base of the tail with 100 μg chicken type II collagen (Sigma) emulsified in complete Freund's adjuvant. Clinical signs of arthritis were assessed visually in the wrist and ankle joints 3 times weekly, using a previously described severity scale: 0 = no arthritis; 1 = 1 inflamed digit; 2 = 2 inflamed digits; 3 = more than 2 digits and footpad inflamed; 4 = all digits and footpad inflamed [17]. Scoring was conducted under blinded conditions for up to 96 days. At day 96 single cell suspensions from lymph nodes (LN) and spleens were restimulated for 6 h with PMA (Sigma; 50 ng/ml) ionomycin (Sigma; 10 ng/ml) and monensin (Biolegend; 1 in 1000) and expression of IFNγ (clone XMG1.2; Biolegend), IL-17 (clone TC11-18H10.1; Biolegend), TNFα (clone MP6-XT22; Biolegend), IL-4 (clone 11B11; Biolegend) and IL-10 (clone JES5-16E3; Biolegend) determined by intracellular flow cytometry.

### 2.3 Total CD4 and naïve CD4^+^ T-cell isolation

Naïve CD4^+^CD44^−^ T-cells from the LNs and spleens of WT and *Ptpn22*^*−/−*^ mice were selected using MACS naïve CD4^+^ negative selection kit according to manufacturer's instructions (Miltenyi Biotech). Total CD4^+^ T-cells from the lymph nodes (LN) and spleens of WT OT-II mice were isolated using CD4^+^ MACS negative selection kit according to manufacturer's instructions (Miltenyi Biotech). Purity of naïve CD4^+^CD62L^+^ T-cells and total CD4^+^ T-cells was determined by flow cytometry to be routinely 90–95%.

### 2.4 Differentiation of primary naïve or total CD4^+^ T cells

Naïve CD4^+^ CD44^lo^ CD62L^+^ T-cells or total CD4^+^ T-cells were resuspended in RPMI-1640 with l-glutamine supplemented with 10% heat-inactivated FBS, β-mercaptoethanol (50 μM), penicillin/streptomycin (100 μg/ml), HEPES (10 mM) and plated at 1 × 10^6^ cells/ml in 48 well flat-bottomed tissue culture plates coated overnight with anti-CD3 (0–2 μg/ml; Biolegend) in the presence or absence of ICAM-1-Fc (0–2 μg/ml; Biolegend). Naïve T-cells were cultured in the presence of soluble anti-CD28 (2 μg/ml; Biolegend) and IL-2 (20 ng/ml) for Th0 conditions, or IL-2 (20 ng/ml), IL-12 (20 ng/ml; Peprotech) and anti-IL-4 (10 μg/ml; Biolegend) for Th1 polarising conditions. IL-2 was added to the culture medium every 48 h to achieve a final concentration of 10 ng/ml. Total CD4^+^ T-cells were cultured at 1 × 10^6^ cells/ml in 96-well flat-bottom plates in the presence or absence of IL-12 (20 ng/ml) and anti-IL-4 (10 μg/ml) for Th1. On day 3 half the media was removed and cells were transferred to a new 96-well plate and media refreshed with IL-2 (20 ng/ml). CD4^+^ T-cell cultures were maintained for 5 days, at which point intracellular cytokine staining for cytokines was performed.

### 2.5 Bone marrow derived dendritic cell (BMDC) culture

BMDC were produced using a protocol adapted from Inaba *et al*. [21]. Bone marrow was flushed from femurs and tibias of WT and *Ptpn22*^−/−^ mice with RPMI-1640 supplemented with l-glutamine (Corning) containing 1% FBS and penicillin/streptomycin (100 μg/ml). Bone marrow cells were incubated in Petri dishes for 30 min at 37 °C in 5% CO_2_ to remove adherent macrophages. Non-adherent progenitor cells were seeded at 1.5 × 10^6^ cells/ml in 24-well tissue culture plates in RPMI-1640 with l-glutamine supplemented with 10% heat-inactivated FBS, β-mercaptoethanol (50 μM), penicillin/streptomycin (100 μg/ml) and 1% murine GM-CSF; GM-CSF was produced from the B78H1/GMCSF.1 cell line. BMDC were cultured for 6 days at 37 °C and 5% CO_2_ and medium was partially replenished on day 3 and fully replaced on day 4. BMDCs were used in functional assays on days 6 and 7. Cells were harvested by washing with PBS containing 2 mM EDTA. At day 6 WT and *Ptpn22*^*−/−*^ immature DC generated were routinely >90% CD11c^+^ determined by flow cytometry.

### 2.6 BMDC phenotype

BMDCs were harvested and stained using anti-mouse CD11c (clone N418; Biolegend), anti-mouse CD11b (clone M1/70; Biolegend), MHC class II I-Ab (clone AF6-120.1; Biolegend), CD86 (clone GL-1; Biolegend), CD40 (clone 3/23; Biolegend), and Zombie Fixable Viability dye (Biolegend) in PBS containing anti-mouse CD16/CD32 (clone 93; Biolegend). Cells were washed with FACS buffer (5% FBS, 1 mM EDTA, 0.01% NaN_3_ in PBS) and fixed using 1% paraformaldehyde (PFA, Electron Microscopy Services) in FACS buffer.

### 2.7 *In vitro* co-cultures

WT and *Ptpn22*^*−/−*^ BMDC were matured overnight in the presence of soluble LPS from *E. coli* at 100 ng/ml (Invivogen). Mature BMDCs were pulsed with OVA_323-339_ (Invivogen) at 1 μg/ml for 4 h at 37 °C. BMDC were harvested, washed and resuspended in RPMI-1640 with l-glutamine supplemented with 10% heat-inactivated FBS, β-mercaptoethanol (50 μM), penicillin/streptomycin (100 μg/ml). CD4^+^ OT-II T-cells were isolated and 2 × 10^7^ cells/ml were labelled with 2 μM CellTrace Violet (CTV, Invitrogen) for 20 min at 37 °C. BMDC were co-cultured with CTV labelled CD4^+^ T-cells at a BMDC:T-cell ratio of 1:2 (5 × 10^4^ BMDC:1 × 10^5^ T-cells) in 96-well round bottom plates for 6 days. On the sixth day of co-culture, 100 μl of complete RPMI was removed and freshly pulsed BMDCs of the same original genotype were added to the culture for a further 48 h (day 8). Proliferation and intracellular cytokine production by OT-II T cells were assessed by flow cytometry and soluble cytokine production determined by immunoassay on days 6 and 8.

### 2.8 Cytokine immunoassays and cell phenotyping

Cell-free co-culture supernatants were harvested at the indicated times. IL-2 and IFNγ were determined by immunoassay. Antibody pairs were purchased from Biolegend. Cytokine levels were determined using streptavidin-europium and enhancement solution (both from Perkin Elmer) and detected on a Victor 1420 multilabel counter (Perkin Elmer). Day 1 or 6 BMDC and OT-II T-cell co-cultures were harvested and stained with anti-CD3ε-FITC (clone 145.2C11; Biolegend), anti-CD4-PerCP (clone RM4-5; Biolegend), anti-CD69 (clone H1.2F3; Biolegend), anti-CD25 (PC61; Biolegend) and Fixable Viability Dye eFluor 506(eBiosciences). Cells were fixed in 1% PFA in PBS. Proliferating and activated T-cell populations were determined by gating on live, singlet, CD3^+^, CD4^+^ cells and by CTV dilution.

### 2.9 Planar supported lipid bilayers

For microscopy CD4^+^ T-cells (1 × 10^6^ cells/ml) were activated for 2 days with mouse anti-CD3/anti-CD28 Dynabeads (1 × 10^6^ beads/ml; Invitrogen) in the presence of 20 ng/ml IL-2. On day 2 beads were magnetically removed and T-cells rested in media in the presence of 20 ng/ml IL-2 for a further 4–6 days. Lipid bilayers were prepared as previously described [22]. 25 × 75-mm glass coverslips were cleaned with Piranha solution (Cyantek Corporation) and adhered to sticky-Slides VI0.4 (Ibidi). Liposomes containing 0.01 mol% biotin-CAP-phosphatidylethanolamine, 97.49% mol% phosphatidylcholine, and 2.5 mol% Ni^2+^-chelating lipids (Avanti Polar Lipids) were placed in to the chambers to form planar bilayers. After blocking for 30 min with 5% casein, 100 μM NiCl_2_, followed by 30 min with fluorescently labelled mouse ICAM-1-12His (200 molecules/μm^2^), and unlabelled streptavidin (5 μg/ml, Sigma), and 30 min with mono-biotinylated Fab' fragments of mouse anti-CD3ε antibodies (5 μg/ml) were added to the chambers. The UCHT1 density was 30 molecules/μm^2^. Flow chambers were warmed to 37 °C and cells added for the indicated time points, prior to fixing with 2% PFA at room temperature for 20 min.

### 2.10 Microscopy

Total internal reflection fluorescence (TIRF) microscopy imaging was performed on an Olympus IX83 motorized TIRF microscopy with a 150×/1.45 NA objective and an Andor IXON ULTRA 512X512 EMCCD camera. For each cell the mean intensity of CD3 and ICAM-1 was calculated by dividing the total fluorescence intensity of the region of interest (ROI) minus background fluorescence of a neighbouring area (measured using the same ROI) and then divided by the area of the ROI.

### 2.11 DC-T cell conjugate assay

LPS (Invivogen; 100 ng/ml) matured WT BMDCs pulsed with OVA_323-339_ (1 μg/ml) were used to activate WT or *Ptpn22*^*−/−*^ T-cells for 6 days. On day 6 a fresh preparation of LPS matured, OVA_323-339_ pulsed (1 μg/ml) WT BMDC were harvested, washed and resuspended at 1 × 10^7^ cells/ml prior to staining with 2 μM CellTrace Far Red (CTFR, Invitrogen). Day 6 activated T-cells were harvested and resuspended at 2 × 10^7^ cells/ml prior to staining with 1 μM CellTrace Violet (CTV, Invitrogen). Staining was performed for 20 min at 37 °C, followed by quenching in culture media for 20 min at 37 °C. 5 × 10^4^ CTFR labelled WT BMDCs and 1 × 10^5^ CTV labelled WT or *Ptpn22*^*−/−*^ OT-II CD4^+^ T-cells (in a total volume of 50 μl) were added to 1.5 ml tubes, centrifuged for 2 min at 500 rpm and incubated at 37 °C for the indicated times. Cells were fixed with 3% PFA in PBS for 15 min at room temperature, transferred to FACS tubes and acquired by flow cytometry using a medium flow rate. Conjugates were identified as CTV^+^ CTFR^+^ cells.

### 2.12 Flow cytometry

Gates were determined by fluorescence minus one (FMO) controls. Cells were acquired using Becton Dickinson Fortessa or FACSCanto II flow cytometers, and data analysed using FlowJo Version 8.7.

### 2.13 Statistical analysis

GraphPad Prism software was used for statistical analysis by unpaired, paired T-test, one-way, or two-way ANOVA with Sidak's post-test (paired or unpaired; two-tails). In arthritis experiments, the area under the curve (AUC) was computed and compared between genotypes by Mann Whitney *U* test. *P* values less than 0.05 were considered significant.

## 3 Results

### 3.1 Th1 effector cells preferentially accumulate in lymphoid organs of *Ptpn22*^*−/−*^ and *Ptpn22*^*619W*^ mice

**Fig. 1:**
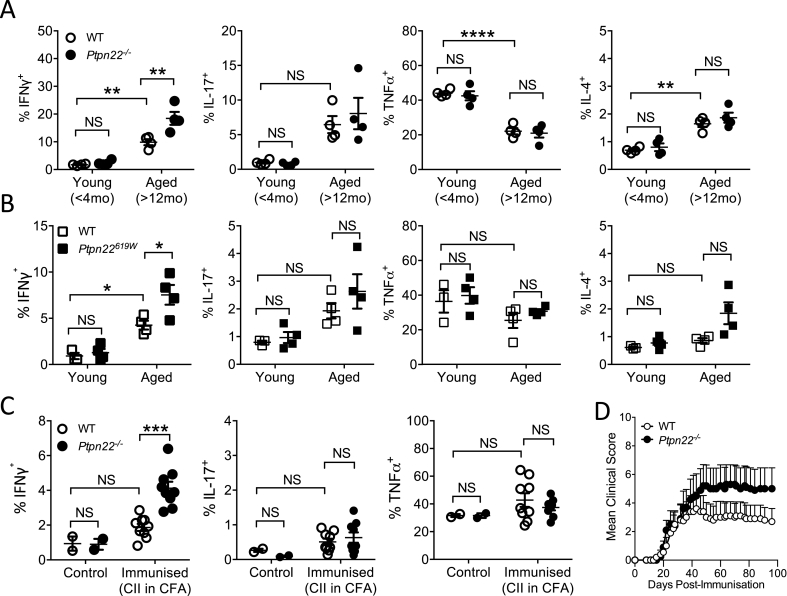
***Ptpn22***^***−/−***^**and *Ptpn22***^***R619W***^**mice accumulate Th1 cells with age or following chronic immune challenge.** Skin draining lymph nodes of <4month (Young) and >12-month-old (Aged) **(A)** WT and *Ptpn22*^*−/−*^ mice or **(B)** WT and *Ptpn22*^*R619W*^ were assessed for intracellular IFNγ, IL-16, TNFα, and IL-4 cytokine production following 6 h PMA, Ionomycin and monensin stimulation, determined by intracellular flow cytometry. N = 4–5 mice per genotype/time point. **(C, D)** For collagen induced arthritis model WT and *Ptpn22*^*−/−*^ mice were immunised with CII in CFA (immunised) and compared to unimmunised mice (control). **(C)** Day 96 lymph nodes were restimulated for 6 h with PMA, ionomycin and monensin for 6 h and expression of IFNγ, IL-17, and TNFα determined by intracellular flow cytometry. **(D)** Mean clinical score of WT and *Ptpn22*^*−/−*^ immunised mice 0–96 days. N = 10 mice/group; bars represent mean + s.e.m. **(A**–**C)** Each point represents an individual mouse, bars represent mean ± s.e.m.; NS = not significant, *p < 0.05, **p < 0.01, ***p < 0.001, ****p < 0.0001 by two-way ANOVA, applying Sidak's multiple comparisons test.

*In vivo*, lymphoid organs of *Ptpn22*^*−/−*^ and *Ptpn22*^*619W*^ mice display an age dependent expansion of CD4^+^ CD44^hi^ CD62L^lo^ effector/memory T-cells and CD4^+^ CD25^+^ FoxP3^+^ regulatory T cells (Tregs) [3,20]. We hypothesised that PTPN22 also regulates the cytokine producing capability of *in vivo* CD4^+^ T-cells. We first assessed the capability of CD4^+^ T-cells from the lymphoid organs of young (<4 months) and aged (>12 months) WT and *Ptpn22*^*−/−*^ mice to produce IFNγ, IL-4, IL-17, and TNFα. As previously reported, *Ptpn22*^*−/−*^ mice exhibited age dependent expansion of CD25^+^Foxp3^+^ regulatory T-cells compared to WT controls (Supplementary Fig. 1A), while WT and *Ptpn22*^*−/−*^ CD4^+^ T-cells from mice <4 months had similar frequencies of IFNγ, IL-17, TNFα and IL-4 producing cells (Fig. 1A, Supplementary Fig. 1B). In comparison, although the frequency of IFNγ^+^ CD4^+^ T cell was increased in aged mice regardless of genotype, aged *Ptpn22*^*−/−*^ mice displayed a significantly expanded population of IFNγ^+^ CD4^+^ T-cells in both the lymph nodes and spleens (Fig. 1A and Supplementary Fig. 1B). Furthermore, mice expressing *Ptpn22*^*W619*^ (the murine orthologue of the human autoimmune associated variant) also displayed a significant age dependent increase in the frequency of IFNγ producing CD4^+^ T-cells compared to *Ptpn22*^*R619*^ (WT) aged-matched controls (Fig. 1B). *Ptpn22* mediated expansion of IFNγ^+^ T-cells appeared specific to IFNγ, as no significant *Ptpn22* dependent changes to the frequencies of IL-17, IL-4 or TNFα^+^ CD4^+^ T cells were observed in either young or aged mice (Fig. 1A and B).

We next assessed if immune challenge of young mice in the context of the collagen induced arthritis (CIA) model was sufficient to reproduce differences in IFNγ^+^ T-cell frequencies according to genotype. Ninety-six days after arthritis induction, spleens and lymph nodes of WT and *Ptpn22*^*−/−*^ mice were pooled and assessed for IFNγ^+^ CD4^+^ T-cells. Following immune challenge, *Ptpn22*^*−/−*^ mice preferentially accumulate IFN-γ producing CD4^+^ T cells when compared to WT (Fig. 1C). Again, we observed that loss of PTPN22 specifically mediated the expansion of IFNγ^+^ CD4^+^ T-cells as no significant changes were observed in IL-17, and TNFα frequencies. In this CIA model, *Ptpn22*^*−/−*^ mice also developed a visible, yet non-significant, increase in paw swelling compared to WT mice (Fig. 1D). Together, these results suggest that, with aging, or in response to immune challenge, IFNγ^+^ T-cells preferentially accumulate in *Ptpn22*^*−/−*^ and *Ptpn22*^*619W*^ mice.

### 3.2 PTPN22 does not regulate CD3 and CD28 mediated IFNγ production

**Fig. 2:**
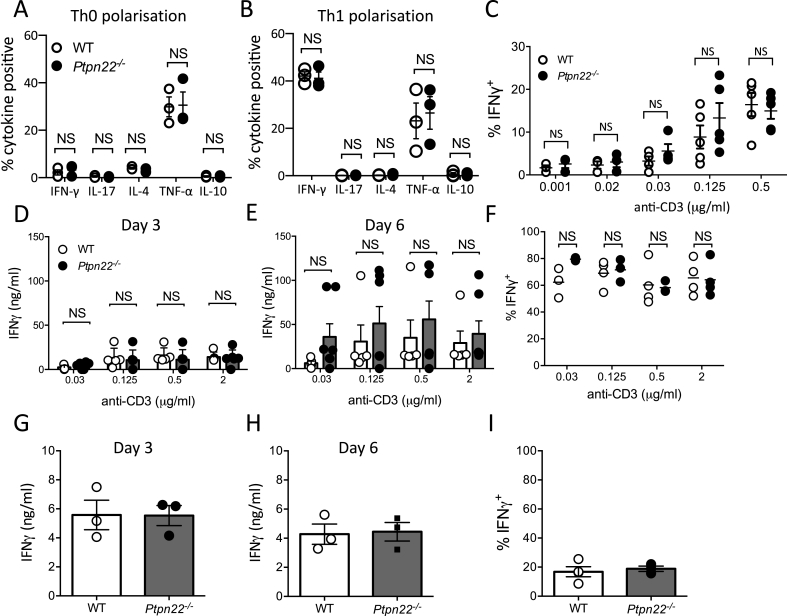
**PTPN22 does not regulate CD3 and CD28 mediated IFNγ production (A, B)** WT and *Ptpn22*^*−/−*^ naïve CD4^+^ T-cells were stimulated via plate bound anti-CD3 and soluble anti-CD28 under **(A)** Th0 (IL-2) and **(B)** Th1 (IL-12, IL-2 and anti-IL-4) polarising conditions. Day 5 T-cells were restimulated for 6 h with PMA, ionomycin and monensin and the proportion of CD3^+^ CD4^+^ IFNγ^+^, IL-17^+^, IL-4^+^, TNFα^+^, or IL-10^+^ cells determined by intracellular flow cytometry. N = 3 independent experiments per genotype **(C)** WT and *Ptpn22*^*−/−*^ naïve T-cells were stimulated via plate bound anti-CD3 (0.001–0.5 μg/ml) and soluble anti-CD28 (2 μg/ml) under Th1 polarising conditions. **(D**–**F)** Total WT and *Ptpn22*^*−/−*^ CD4^+^ T-cells were stimulated via plate bound anti-CD3 (0.03–2 μg/ml) and soluble anti-CD28 (2 μg/ml) under Th1 polarising conditions. Cell free supernatants at **(D)** day 3 and **(E)** day 5 were assessed for IFNγ by ELISA. **(F)** Day 5 T-cells were restimulated for 6 h with PMA, ionomycin and monensin and the proportion of CD3^+^ CD4^+^ IFNγ^+^ cells determined by intracellular flow cytometry. **(G**–**I)** Total WT and *Ptpn22*^*−/−*^ CD4^+^ T-cells were stimulated with plate bound anti-CD3 (2 μg/ml) and soluble anti-CD28 (2 μg/ml) under non-polarising conditions. Cell free supernatants at **(G)** day 3 and **(H)** day 5 were assessed for IFNγ by ELISA. **(I)** Day 5 T-cells were restimulated for 6 h with PMA, ionomycin and monensin and the proportion of CD3^+^ CD4^+^ IFNγ^+^ cells determined by intracellular flow cytometry. (**A-I)** N = 3–5 per group with each data point representing T-cells isolated from an individual mouse, bars represent mean ± s.e.m.; NS = not significant determined by un-paired T-test.

We next sought to investigate *in vitro* the pathways regulated by PTPN22 that might account for expansion of IFNγ^+^ T-cells *in vivo*. Multiple T-cell intrinsic and extrinsic factors regulate the capability of CD4^+^ T cells to produce IFNγ and differentiate into Th1 cells. First, we assessed if PTPN22 was capable of regulating naïve CD4^+^ T-cell differentiation towards a Th1 phenotype in a T-cell intrinsic manner. To address this question WT and *Ptpn22*^*−/−*^ CD4^+^ naïve T cells were stimulated under polarising conditions in the presence of anti-CD3 and anti-CD28, with IL-2 alone (Th0) or IL-2 with IL-12 and anti-IL-4 (Th1). At day six following restimulation with PMA and ionomycin, we observed that under both Th0 (Fig. 2A) or Th1 (Fig. 2B) polarising conditions there was no difference in the proportion of IFNγ, IL-10, IL-4, IL-17, or TNFα^+^ T-cells generated from naïve WT or *Ptpn22*^*−/−*^ T-cells. Previous investigations have revealed that PTPN22 regulates TCR signalling thresholds, and that reduced TCR signal strength, conferred by low affinity antigen, enhances IFNγ production by CD8^+^ T-cells [13]. As such we assessed if reduced levels of anti-CD3 stimulation would reveal differences in the capability of naïve *Ptpn22*^*−/−*^ T-cells to produce IFNγ. However, titrating anti-CD3 revealed no significant differences between the frequency of IFNγ^+^ T-cells generated from WT or *Ptpn22*^*−/−*^ naïve CD4^+^ T-cells (Fig. 2C). Since loss of PTPN22 function predominantly effects the activation of effector/memory T-cells rather than naïve T-cells, we assessed if the differentiation of total (naïve and effector/memory) CD4^+^ T-cells towards a Th1 phenotype would be altered in *Ptpn22*^*−/−*^ mice. Total CD4^+^ T-cells were stimulated with a range of plate bound anti-CD3 concentrations (0.03–2 μg/ml) in the presence of soluble anti-CD28, anti-IL-4 and IL-12. However, even at reduced anti-CD3 concentrations no PTPN22 dependent differences were observed in the frequency of IFNγ^+^ CD4 T-cells or the secretion of IFNγ (Fig. 2D–F). When culturing total CD4^+^ T-cells under IFNγ polarising conditions we observed a very high frequency of IFNγ^+^ cells (Fig. 2F, 60–80%), we therefore sought to exclude the possibility that the presence of IL-12 was masking a PTPN22 mediated regulation of anti-CD3/anti-CD28 stimulation induced IFNγ by performing the cultures in the absence of IL-12. However, despite the reduced frequency of IFNγ^+^ cells generated in the absence of IL-12, no PTPN22 dependent differences in the expansion of IFNγ^+^ CD4^+^ T-cells was observed (Fig. 2G–I). These data indicate that PTPN22 does not negatively regulate CD3/CD28 mediated differentiation of IFNγ^+^ CD4 T-cells.

### 3.3 PTPN22 negatively regulates ICAM-1-LFA-1 dependent T-cell synapse formation and IFNγ secretion

**Fig. 3:**
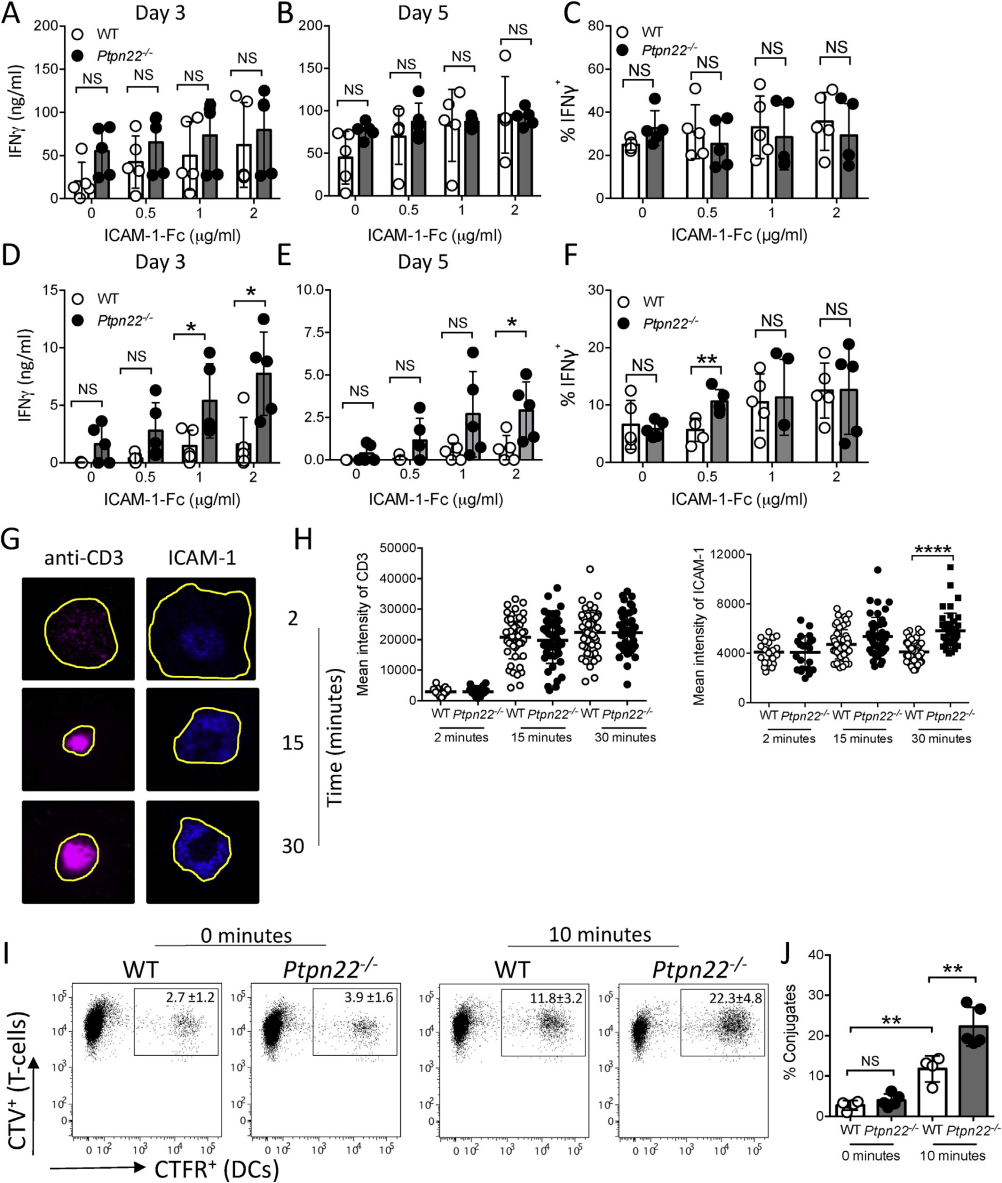
**PTPN22 negatively regulates ICAM-1-LFA-1 dependent T-cell synapse formation and IFNγ secretion. (A**–**C)** WT and *Ptpn22*^*−/−*^ CD4^+^ T-cells were stimulated via plate bound anti-CD3 and ICAM-1-Fc in the presence of IL-12 and anti-IL-4. Cell free supernatants at **(A)** day 3 and **(B)** day 5 were assessed for IFNγ by ELISA. **(C)** Day 5 T-cells were restimulated for 6 h with PMA, ionomycin and monensin and the proportion of CD3^+^ CD4^+^ IFNγ^+^ cells determined by intracellular flow cytometry. **(D**–**F)** WT and *Ptpn22*^*−/−*^ CD4^+^ T-cells were stimulated via plate bound anti-CD3 and ICAM-1-Fc in the presence of anti-IL-4 but the absence of IL-12. Cell free supernatants at **(D)** day 3 and **(E)** day 5 were assessed for IFNγ by ELISA. **(F)** Day 5 T-cells were restimulated for 6 h with PMA, ionomycin and monensin and the proportion of CD3^+^ CD4^+^ IFNγ^+^ cells determined by intracellular flow cytometry. **(G, H)** Immune synapse formation by WT and *Ptpn22*^*−/−*^ CD4^+^ T-cells following incubation on planar supported lipid bilayers containing anti-CD3 and ICAM-1. **(G)** representative example of CD3 and ICAM-1 distribution over time within a WT T-cell immune synapse. **(H)** For each cell the mean intensity of CD3 and ICAM-1 was calculated by dividing the total fluorescence intensity of the region of interest (ROI, shown by yellow outline) by the area of the ROI. >50 cells/group, representative of >3 independent experiments. Bars represent mean ****p < 0.0001 by one-way ANOVA. **(I, J)** Conjugates were formed between activated CTV labelled WT and *Ptpn22*^*−/−*^ OT-II CD4^+^ T-cells and CFTR labelled OVA_323-339_ pulsed WT BMDC. CTV^+^CFTR^+^ conjugates were determined by flow cytometry at 0 and 10 min at 37 °C. **(I)** representative flow cytometry plots, quantified in **(J)** percentage of CD4^+^ T-cells in conjugates. N = 4–5 independent experiments per group. Bars represent mean ± s.d; NS = not significant, *p < 0.05, **p < 0.01 by two-way ANOVA, applying Sidak's multiple comparisons test. (For interpretation of the references to colour in this figure legend, the reader is referred to the Web version of this article.)

LFA-1 is a potent costimulatory molecule capable of enhancing TCR dependent IFNγ secretion by T-cells [17]. PTPN22 acts as a negative regulator of LFA-1 dependent signalling, adhesion and migration [5,20]. We therefore sought to determine whether LFA-1 mediated expansion of IFNγ^+^ T-cells was regulated by PTPN22. WT or *Ptpn22*^*−/−*^ CD4^+^ T-cells were stimulated with plate bound anti-CD3 and a range of ICAM-1-Fc concentrations (0–2 μg/ml) for 3 or 5 days in the presence (Fig. 3A–C) or absence of IL-12 (Fig. 3D–F). In the presence of IL-12 we again observed no difference in the capability of WT or *Ptpn22*^*−/−*^ T-cells to secrete IFNγ (Fig. 3A–C). Interestingly however, in the absence of IL-12 we observed that *Ptpn22*^*−/−*^ T-cells had an enhanced capability to secrete IFNγ in comparison to WT T-cells over multiple ICAM-1 concentrations (Fig. 3D–E). Previous investigations have noted that LFA-1 stimulation predominantly regulates T-cell secretion of IFNγ rather than intracellular expression of IFNγ [18]. Our data supports this as more IFNγ was detected from *Ptpn22*^*−/−*^ cell cultures by ELISA, whereas the proportion of IFNγ^+^ cells was unchanged (Fig. 3D–F), in contrast to anti-CD3/anti-CD28 stimulation which modulated neither IFNγ secretion nor intracellular expression (Fig. 2D–F). We previously observed that *Ptpn22*^*−/−*^ T-cells display enhanced phosphorylation of multiple kinases downstream of LFA-1 [5], which may account for the increased LFA-1 mediated IFNγ secretion in *Ptpn22*^*−/−*^ T-cells. LFA-1 function is controlled by both affinity and avidity [23] following TCR mediated inside-out signalling. The TCR accumulates at the centre of the immunological synapse in extracellular vesicles that activate antigen-presenting cells [24]. LFA-1 forms a ring of densely packed clusters that engage ICAM-1 and mediate dynamic adhesion [25]. Over time we observed that *Ptpn22*^*−/−*^ CD4^+^ T-cells incubated on planar supported lipid bilayers containing anti-CD3 and ICAM-1 displayed an enhanced density of ICAM-1 at the immune synapse compared to WT T-cells, indicative of enhanced LFA-1 interaction with ICAM-1 in *Ptpn22*^*−/−*^ T cells (Fig. 3G and H). By contrast densities of anti-CD3 were not affected. To further test the functional effects of enhanced LFA-1/ICAM-1 interactions during immune synapse formation, we examined T cell-DC conjugate formation. Within 10 min *Ptpn22*^*−/−*^ T-cells were capable of forming more conjugates with WT BMDC, as compared to WT T-cells (Fig. 3I and J). We conclude that enhanced LFA-1 responsiveness, manifested by increased densities of LFA-1-ICAM-1 at the immune synapse and enhanced conjugate formation, could be responsible for the expansion of IFNγ^+^ T-cells.

### 3.4 PTPN22 regulates the capability of BMDC to induce Th1 responses

**Fig. 4:**
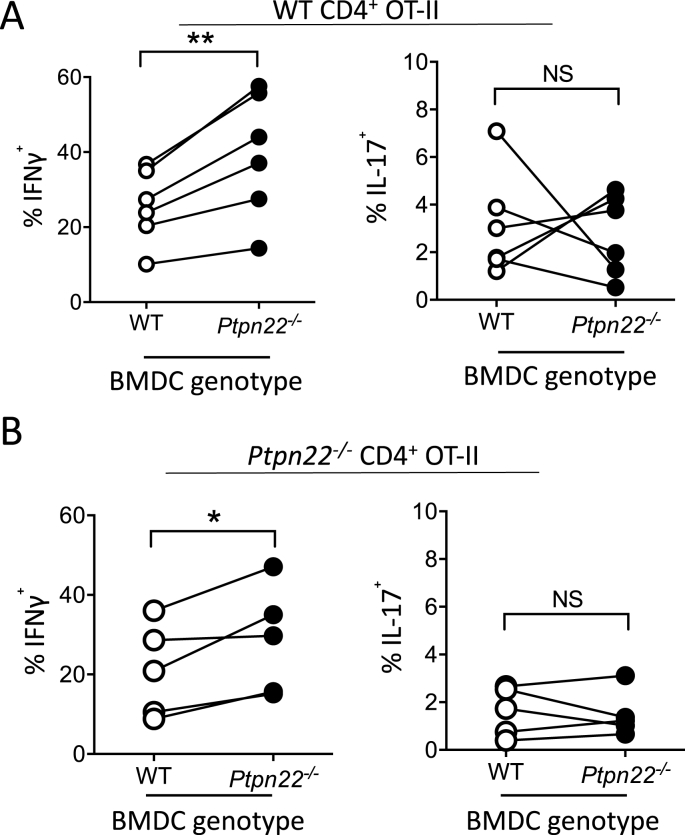
***Ptpn22***^***−/−***^**BMDC are able to promote Th1 responses. (A)** Day 6 WT and *Ptpn22*^*−/−*^ LPS and OVA_323-339_ pulsed BMDC were co-cultured with WT CD4^+^ OT-II T-cells for 6 days. At day 6, T-cells were restimulated with a fresh preparation of WT or *Ptpn22*^−/−^ BMDC for 48 h and then restimulated for 6 h with PMA, ionomycin and monensin and the proportion of CD3^+^ CD4^+^ IFNγ^+^ or IL-17^+^ cells was determined by intracellular flow cytometry. **(B)** Day 6 WT and *Ptpn22*^*−/−*^ LPS and OVA_323-339_ pulsed BMDC were co-cultured with *Ptpn22*^*−/−*^ CD4^+^ OT-II T-cells for 6 days. At day 6 T-cells were restimulated with a fresh preparation of WT or *Ptpn22*^−/−^ BMDC and then restimulated for 6 h with PMA, ionomycin and monensin and the proportion of CD3^+^ CD4^+^ IFNγ^+^ or IL-17^+^ cells determined by intracellular flow cytometry. N = 6–7 per group with each data point representing an individual WT or *Ptpn22*^*−/−*^ BMDC preparation, points are paired by the OT-II responding T-cells. NS = not significant, *p ≤ 0.05, **p ≤ 0.01 determined by paired T-test.

*In vivo* antigen presenting cells (APCs) mediate the activation and differentiation of T-cells. Recent investigations have revealed a role for PTPN22 in DC activation [6,7,10,26]. We therefore assessed if PTPN22 might also regulate the expansion of Th1 responses by modulating APC mediated T-cell activation. Bone marrow derived dendritic cells (BMDCs) from WT and *Ptpn22*^*−/−*^ mice were generated with GM-CSF and matured overnight with LPS prior to pulsing for the final 4 h with OVA_323-339_. In contrast to previously described data, *Ptpn22* was not found to influence the maturation of either immature or LPS matured BMDCs (Supplementary Fig. 3A) [10]. Matured WT or *Ptpn22*^*−/−*^ BMDC were co-cultured with CTV labelled WT CD4^+^ OT-II cells for 6 days and then T-cells re-challenged for a further 48 h with a fresh preparation of LPS and OVA_323-339_ pulsed BMDC of the same WT or *Ptpn22*^*−/−*^ genotype. We compared the capability of the WT and *Ptpn22*^*−/−*^ LPS matured BMDCs to induce OT-II T-cell proliferation and inflammatory cytokine production. Although no difference in T-cell proliferation was noted (Supplementary Fig. 3B), we found that stimulation with *Ptpn22*^*−/−*^ BMDCs significantly enhanced the proportion of IFNγ^+^ OT-II T-cells compared to OT-II cells co-cultured with WT BMDCs (Fig. 4A). Furthermore, as observed *in vivo* the effects of T-cell co-culture with *Ptpn22*^*−/−*^ BMDC appeared to be specific to IFNγ production, as IL-17 responses were not dependent on BMDC PTPN22 expression (Fig. 4A). In addition, we found that lacking *Ptpn22* in both BMDC and in CD4^+^ OT-II T-cells also resulted in enhanced Th1 responses, though the proportion of IFNγ^+^ cells was not further potentiated beyond that of *Ptpn22*^*−/−*^ BMDC co-cultured with WT OT-II T-cells (Fig. 4B). These data indicate that enhanced Th1 responses observed in *Ptpn22*^*−/−*^ mice may also be in part dependent on an enhanced capability of APCs to induce Th1 activation.

### 3.5 *Ptpn22*^*−/−*^ BMDC do not regulate Th1 responses via altered cytokine secretion

**Fig. 5:**
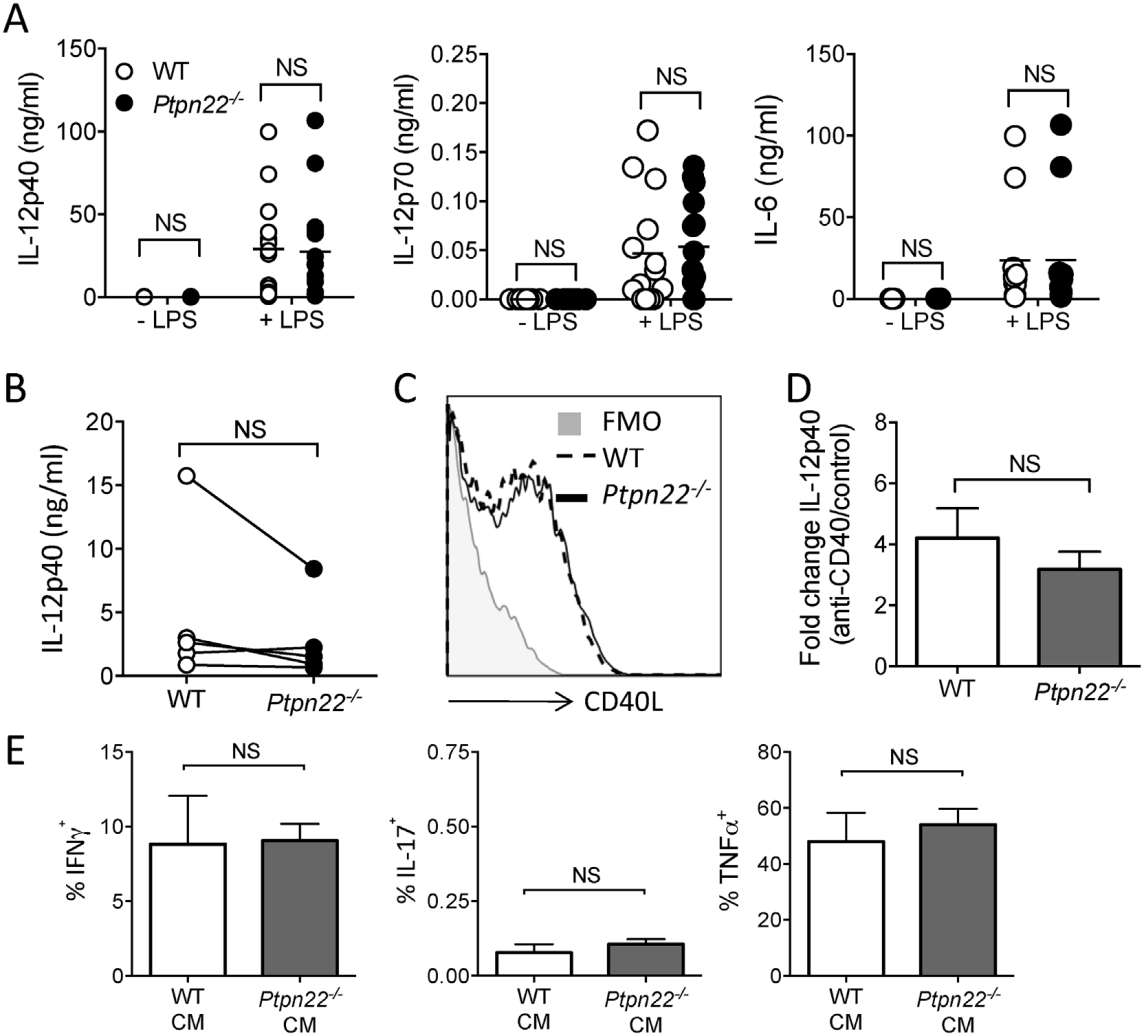
**Potentiated Th1 responses induced by *Ptpn22***^***−/−***^**BMDC are not explained by altered production of a secreted factor. (A)** Day 6 WT and *Ptpn22*^*−/−*^ BMDC were pulsed for 24 h in the presence or absence of LPS. Cell free supernatants were assessed for IL-12p40, IL-12p70 and IL-6 by cytokine specific immunoassay. N = 11–15 independent experiments per group. NS = not significant determined by unpaired T-test. **(B)** Day 6 WT and *Ptpn22*^*−/−*^ LPS and OVA_323-339_ pulsed BMDC were co-cultured with cell trace violet (CTV) labelled WT CD4^+^ OT-II T-cells for 24 h and cell free supernatants were assessed for IL-12p40 by immunoassay. N = 6–7 per group with each data point representing an individual WT or *Ptpn22*^*−/−*^ BMDC preparation, points are paired by the OT-II responding T-cells. **(C)** Day 6 WT and *Ptpn22*^*−/−*^ LPS and OVA_323-339_ pulsed BMDC were co-cultured with CTV labelled WT CD4^+^ OT-II T-cells for 24 h. Cell surface expression of CD40L on CD3^+^ CD4^+^ T-cells was determined by flow cytometry. One representative flow plot of 3 independent experiments; T-cells co-cultured with WT BMDC (dashed line) or *Ptpn22*^*−/−*^ BMDC (solid line) **(D)** LPS pulsed WT and *Ptpn22*^*−/−*^ BMDC were stimulated for 24 h with plate bound anti-CD40 and cell free supernatants were assessed for IL-12p40 by immunoassay. Data are depicted as in IL-12p40 fold change from PBS control to anti-CD40. N = 4 independent experiments; bars represent mean ± s.d. **(E)** WT OT-II T-cells were cultured for 6 days on anti-CD3 and ant-CD28 in the presence of 50% conditioned media obtained from day 6 WT or *Ptpn22*^*−/−*^ BMDC:WT OT-II co-cultures. At day 6 of culture in the presence of conditioned media T-cells were restimulated for 6 h with PMA, ionomycin and monensin and the proportion of CD3^+^ CD4^+^ IFNγ^+^, IL-17^+^, or TNFα^+^ cells determined by intracellular flow cytometry. N = 3 independent experiments; bars represent mean ± s.d. NS = not significant, *p ≤ 0.05, **p ≤ 0.01 determined by unpaired T-test.

To explore how *Ptpn22*^*−/−*^ BMDC were capable of inducing enhanced Th1 responses we assessed if LPS stimulation of WT and *Ptpn22*^*−/−*^ BMDC resulted in differing Th1 skewing cytokine profiles. IL-12 is known to promote Th1 responses and previous investigations have revealed that *Ptpn22*^*R619W*^ results in enhanced IL-12 production from BMDC following LPS stimulation. We therefore assessed the secretion of both the IL-12p40 subunit (common to IL-12 and IL-23) and IL-12p70. However, we observed no significant PTPN22 dependent difference in the secretion of these factors in the presence or absence of LPS stimulation (Fig. 5A). Ligation of CD40 on BMDC by CD40L expressed on activated T-cells results in enhanced BMDC cytokine production [18]. To assess if day 6 T-cells from *Ptpn22*^*−/−*^ BMDC co-cultures were able to induce enhanced IL-12p40 secretion through CD40 ligation, we measured IL-12p40 secretion in the first 24 h of the secondary co-culture. Once again, we observed that there was no PTPN22 dependent difference in IL-12p40 secretion (Fig. 5B). In support of this, no difference in OT-II T-cell surface expression of CD40L was observed following primary culture with either WT or *Ptpn22*^*−/−*^ BMDC (Fig. 5C). Furthermore, we found that following culture of LPS activated WT or *Ptpn22*^*−/−*^ BMDC on plate bound anti-CD40 no PTPN22 dependent difference in IL-12p40 secretion could be detected (Fig. 5D). Finally, to assess if any unidentified BMDC secreted factor/s was capable of mediating the enhanced Th1 response following co-culture with *Ptpn22*^*−/−*^ BMDC we performed a conditioned media experiment. OT-II T-cells were cultured on anti-CD3 and anti-CD28 for 6 days in the presence or absence of 50% conditioned media from day 6 OT-II T-cell co-cultures with either WT or *Ptpn22*^*−/−*^ BMDCs. However, we also observed that there was no PTPN22 dependent difference in the generation of either IFNγ, IL-17 or TNFα^+^ cells following culture with WT or *Ptpn22*^*−/−*^ conditioned media (Fig. 5E). These data indicated that the expanded Th1 responses observed in *Ptpn22*^*−/−*^ co-cultures were unlikely to be explained by a DC derived secreted factor.

### 3.6 DC expression of PTPN22 regulates Th1 differentiation through an LFA-1 contact dependent mechanism

**Fig. 6:**
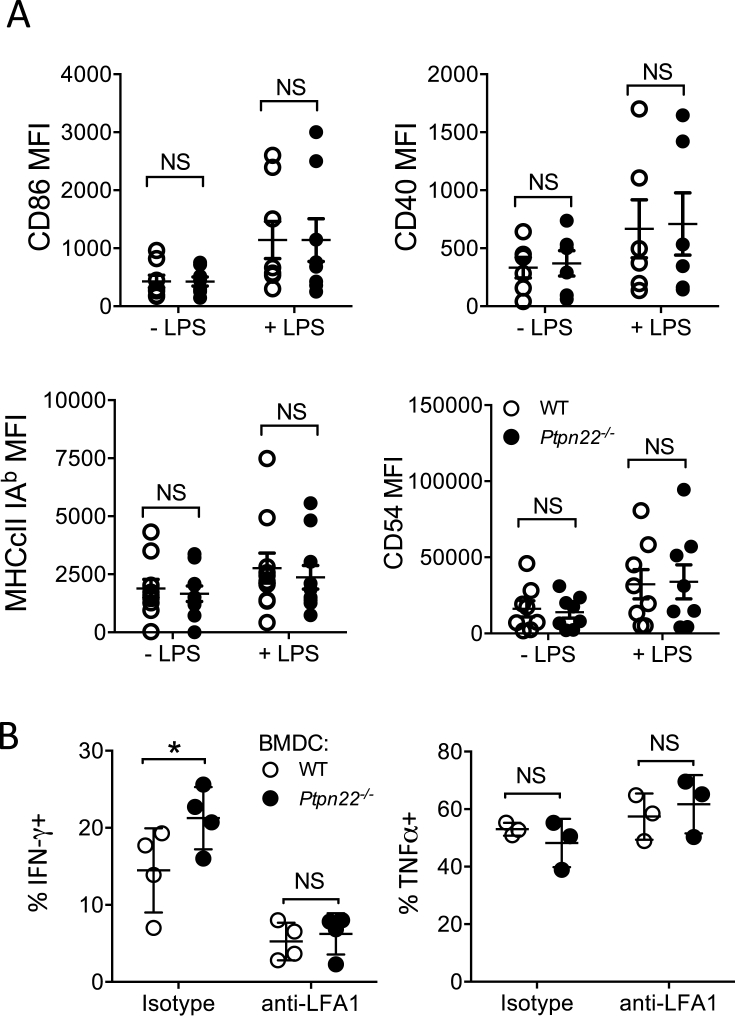
***Ptpn22***^***−/−***^**BMDC promote Th1 responses in an LFA-1 dependent manner. (A)** Day 6 WT and *Ptpn22*^*−/−*^ BMDC were pulsed for 24 h in the presence or absence of LPS. Cell surface expression of maturation markers was determined by flow cytometry. Median Fluorescent intensity (MFI) of CD86, CD40, MHCcII IA^b^, and CD54 (ICAM-1). N = 6–10 independent experiments; bar represents mean ± s.e.m, NS = not significant determined by unpaired T-test. **(B)** Day 6 WT and *Ptpn22*^*−/−*^ LPS and OVA_323-339_ pulsed BMDC were co-cultured with cell trace violet (CTV) labelled WT CD4^+^ OT-II T-cells for 6 days in the presence or absence of anti-LFA1 or isotype control. At day 6 cells were T-cells were restimulated with a fresh preparation of WT or *Ptpn22*^−/−^ BMDC for 48 h and then restimulated for 6 h with PMA, ionomycin and monensin and the proportion of CD3^+^ CD4^+^ IFNγ^+^ or TNFα^+^ cells was determined by intracellular flow cytometry. N = 4 independent experiments; bar represents mean ± s.d, NS = not significant, *p ≤ 0.05, determined by unpaired T-test.

We next assessed if a contact dependent mechanism may explain enhanced Th1 responses following co-culture with *Ptpn22*^*−/−*^ BMDC. As described above, PTPN22 is a negative regulator of LFA-1 signalling in T-cells and LFA-1 signalling regulates Th1 responses (Fig. 3). We hypothesised that enhanced Th1 responses elicited by co-culture with *Ptpn22*^*−/−*^ BMDC might be due in part to enhanced LFA-1 signals. Firstly, we observed no difference in the cell surface expression of ICAM-1 (CD54), CD40, CD86 or MHCII on WT or *Ptpn22*^*−/−*^ BMDC following LPS maturation (Fig. 6A and Supplementary Fig. 3A). WT OT-II T-cells were co-cultured with WT or *Ptpn22*^*−/−*^ BMDC (as in Fig. 4) for 8 days in the presence of either neutralising anti-LFA-1 or isotype control and the proportion of intracellular IFNγ^+^ OT-II T-cells assessed. We observed that in the presence of the isotype control OT-II T-cells co-cultured with *Ptpn22*^*−/−*^ BMDC generated a significantly enhanced proportion of IFNγ^+^ cells, as in Fig. 4A (Fig. 6B). Interestingly, we observed that the enhanced IFNγ^+^ phenotype conferred by *Ptpn22*^*−/−*^ BMDC was lost when OT-II T-cells were cultured in the presence of anti-LFA-1 (Fig. 6B), indicating that the difference in co-culture induced Th1 responses may be, at least in part, due to an LFA-1 dependent mechanism.

Taken together, our data demonstrate that IFNγ^+^ CD4^+^ T-cells are specifically expanded *in vivo* with age or following an inflammatory immune response. Our data indicate that perturbations to ICAM-1-LFA-1 signalling conferred by the absence of PTPN22 in either T-cells or DCs potentiates IFNγ secretion by CD4 T-cells, thereby contributing to the expansion of IFNγ^+^ CD4^+^ T-cells observed *in vivo*.

## 4 Discussion

Here, we describe how PTPN22 contributes to the expansion of Th1 cells *in vivo* in aged *Ptpn22*^*−/−*^ and *Ptpn22*^*619W*^ mice or following a repeated immune challenge in *Ptpn22*^*−/−*^ mice by regulating LFA-1 dependent interactions. We propose that PTPN22 operates in both a T-cell intrinsic and extrinsic manner to regulate LFA-1 dependent Th1 cell responses, in turn influencing the overall signalling threshold over time to promote IFNγ production (A schematic, summarising these mechanistic features, is provided in Supplementary Fig. S4.).

As observed in previous investigations, we found that 2-4-month-old *Ptpn22*^*−/−*^ mice have similar proportions of IFNγ^+^ T-cells, but an expanded repertoire of regulatory T-cells as mice age [14,20]. However, we noted that as *Ptpn22*^*−/−*^ mice age the expansion of regulatory T-cells coincides with an expansion of Th1 cells. These data indicate that with aging or following certain types of chronic immune challenge the balance of the immune response is potentially altered in favour of a more inflammatory phenotype. The onset of many autoimmune diseases tends to occur in the 5^th^-6th decades of life [25]. Therefore, our data may provide some explanation as to how perturbations to PTPN22 promote autoimmunity later in life, following an alteration of ICAM-1-LFA-1 responsiveness over time, which accumulates to promote the expansion of inflammatory IFNγ responses with aging.

Although we observed that CD3/CD28 mediated induction of CD4^+^ Th1 responses are intact in *Ptpn22*^*−/−*^ T-cells even under conditions of low avidity stimulation [14], we cannot exclude the possibility that under conditions of low affinity TCR stimulation CD4^+^ Th1 responses may also be regulated in a similar manner to CD8^+^ T-cells activated with low affinity altered peptide ligands [13]. An important finding was that anti-CD3 and LFA-1 stimulation of *Ptpn22*^*−/−*^ T-cells leads to increased IFNγ responses. These data correlated with an enhanced ability of *Ptpn22*^*−/−*^ T-cell LFA-1 to engage ICAM-1, which manifests downstream through increased T cell-DC conjugates. Our data indicate that PTPN22 acts as a phosphatase that fine-tunes T-cell immune synapse signals with functionally important consequences. Multiple studies have demonstrated that perturbations to PTPN22 can alter the balance of the immune response. For example, overexpression of PTPN22 resulted in attenuated Th1 differentiation at low strength TCR stimulation and protected mice from a model of diabetes [27]. Further studies have demonstrated that PTPN22 is capable of regulating the Th17 bias in mannan immunised SKG mice towards a Th1/Treg phenotype, protecting the mice from arthritis [16]. Although *Ptpn22*^*−/−*^ mice on the C57BL/6 background do not spontaneously develop autoimmunity (due to the enhanced immunosuppressive function of expanded regulatory T cells), our data indicate that over time there is a concomitant expansion in Th1 cells. The expansion of Th1 cells in order to overcome the immunosuppressive capabilities of *Ptpn22*^*−/−*^ regulatory T-cells is likely to be a crucial checkpoint beyond which autoimmune disease becomes manifest.

Recent studies have indicated that PTPN22 also indirectly regulates T-cell responses via regulation of antigen presenting cell function. Here, we observed that PTPN22 regulates BMDC induced Th1 responses via a contact dependent mechanism, which most likely occurs, at least in part, via ICAM-1-LFA-1 engagement. Similar to our previous investigation [26], but in contradiction to a study assessing BMDC from *Ptpn22*^*619W*^ mice [10], PTPN22 was not found to regulate LPS induced expression of cell surface maturation markers or secreted cytokines by BMDC. The reasons for these discrepancies may point to the subtle role that PTPN22 plays in fine tuning signalling thresholds, such that differences in stimulation dosing modulates if, and how, PTPN22 regulates a signalling cascade. Indeed, our data indicate that PTPN22 regulates LPS matured BMDC induced Th1 induction only over multiple rounds of T-cell stimulation; potentially offering an explanation as to why expanded Th1 responses occur only with aging or over long durations of immune challenge.

The data presented here are limited to murine *Ptpn22*^*−/−*^ and *Ptpn22*^*619W*^ models. However, recent investigations indicate that common pathways operate between species. Supplementary Table 1 highlights studies reported to date that have assessed how the murine and human PTPN22 variants regulate Th1/LFA-1 responses. The evidence indicates that in both mouse and man IFNγ^+^ T-cells are expanded, though the conditions through which expansion is observed may differ. Several groups have reported an association between the PTPN22-620W variant, increased pErk signalling and IFNγ expression [5,28,29]; our murine data provides new mechanistic insights as to how this may be occurring in man.

## 5 Conclusions

Here we provide evidence to suggest that PTPN22 negatively regulates LFA-1 dependent production of IFNγ producing Th1 cells via T-cell intrinsic and extrinsic mechanisms. These data indicate that PTPN22 plays important role in regulating the thresholds of signalling as immune responses mature over time and the impact that this could confer on the resulting immune response.

## Contributors

CSB, FC, GHC performed experiments, analysed data and contributed to the writing of the paper. DD and MLD provided planar supported lipid bilayer assays, analysed data and contributed to the writing of the paper. ST performed CIA experiments and contributed to analysis. XD, DJR, and RZ developed mouse models and contributed to the writing of the paper. APC conceived the project, analysed data and contributed to the writing the manuscript. HAP performed experiments, analysed data and wrote the manuscript. All authors reviewed the manuscript.

## Funding

This research was supported by Arthritis Research UK grants 20218 (awarded to H.A.P and A.P.C), 20525 (awarded to G.H.C, R.Z and A.P.C), Wellcome Trust Investigator Award 096669AIA (awarded to R.Z) and Principal Research Fellowship 100262 (awarded to MLD), and NIH contracts DP3-DK097672 and DP3-DK111802 (to D.J.R). Additional support was provided by the Children's Guild Association Endowed Chair in Pediatric Immunology and the Benaroya Family Gift Fund (to D.J.R.). This work was also supported by infrastructure funded by the National Institute for Health Research (NIHR) BioResource Clinical Research facility and Biomedical Research Centre based at Guy's and St. Thomas' NHS Foundation Trust and King's College London (reference: guysbrc-2012-17). The content is solely the responsibility of the authors and does not necessarily represent the official views of the National Institutes of Health.

## Conflict of interests

The authors have no competing or financial interests to declare.
