# Supplementary material for: Protein tyrosine phosphatase PTPN22 regulates LFA-1 dependent Th1 responses
Source: J Autoimmun. 2018 Nov;94:45–55. doi: 10.1016/j.jaut.2018.07.008 (PMC6198113; doi:10.1016/j.jaut.2018.07.008)
Supplement: Multimedia component 1 [file mmc1.pdf]

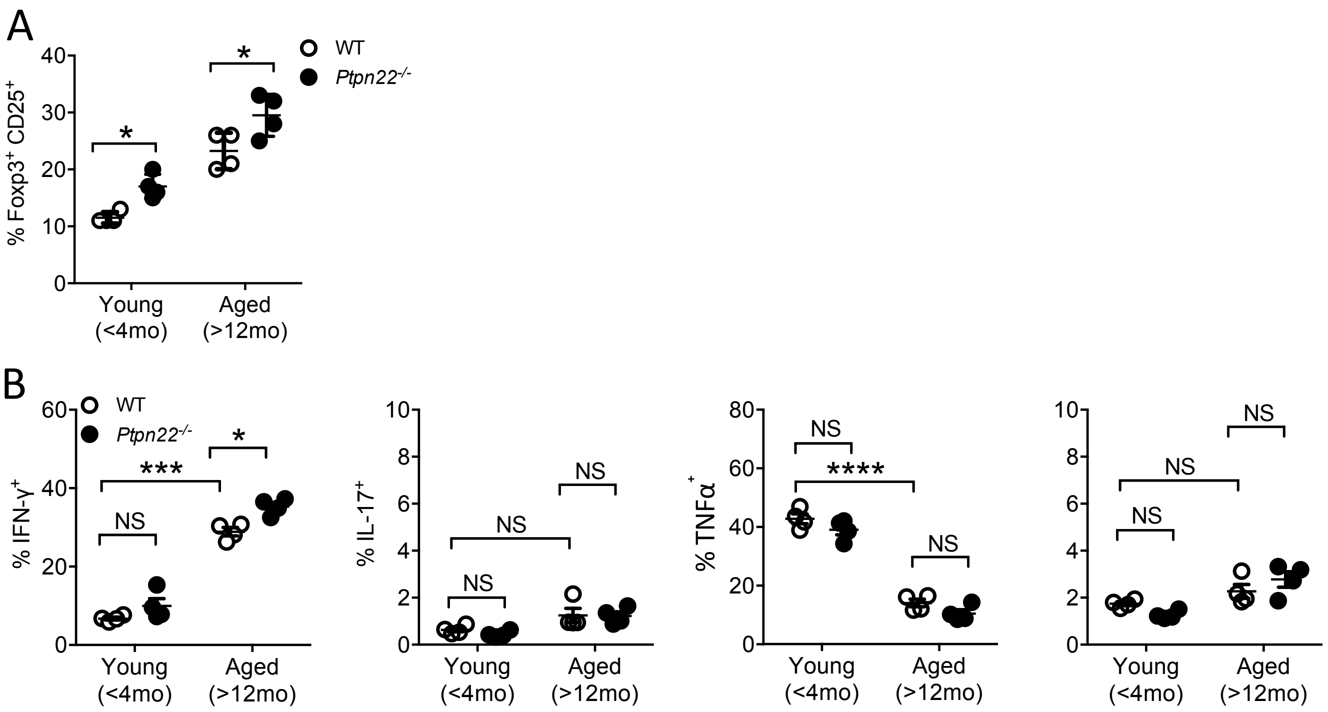

Supplementary figure S1. *Ptpn22*$^{-/-}$ accumulate Th1 cells in the spleen with age. (A) Spleens of <4month and >12 month old T and *Ptpn22*$^{-/-}$ mice were assessed for CD4^{+} Foxp3^{+}CD25^{+} Tregs by intracellular flow cytometry **(B)** Spleens of <4month and >12 month old WT and *Ptpn22*$^{-/-}$ mice were assessed for intracellular IFNγ, IL-17, TNFα, and IL-4 cytokine production following 6 hours PMA, ionomycin and monensin stimulation, determined by intracellular flow cytometry. N=4-5 mice per genotype/time point. NS = not significant, *p<0.05,, ***p<0.001, ****p<0.0001 by two-way ANOVA, applying Sidak's multiple comparisons test.

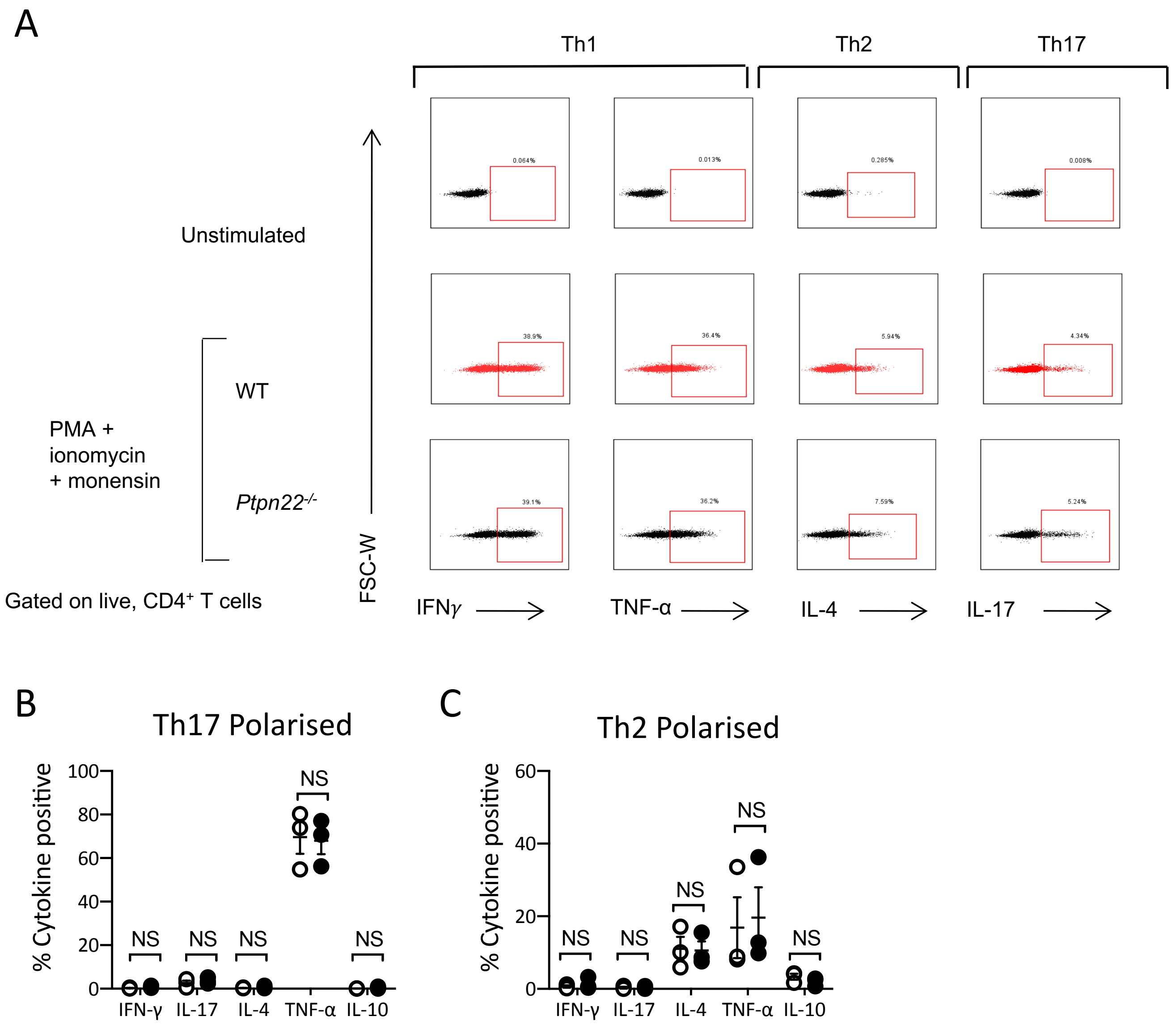

Supplementary figure S2. T-cell activation via CD3 and CD28 does not induce enhanced Th17 or Th2 responses from *Ptpn22$^{-/-}$* T-cells. (A - C) WT and *Ptpn22$^{-/-}$* naïve $CD4^+$ T-cells were stimulated via plate bound anti-CD3 and soluble anti-CD28 under Th1, Th2, or Th17 polarising conditions. Day 5 T-cells were restimulated for 6 hours with PMA, ionomycin and monensin and the proportion of $CD3^+$ $CD4^+$ $IFN\gamma^+$, $IL\text{-}17^+$, $IL\text{-}4^+$, TNFα cells determined by intracellular flow cytometry. **(A)** Representative flow cytometry staining of cytokine producing cells; Th1 replicates shown in Fig 2A. **(B)** Th2 polarisation **(C)** Th17 polarisiation N=3 independent experiments per genotype

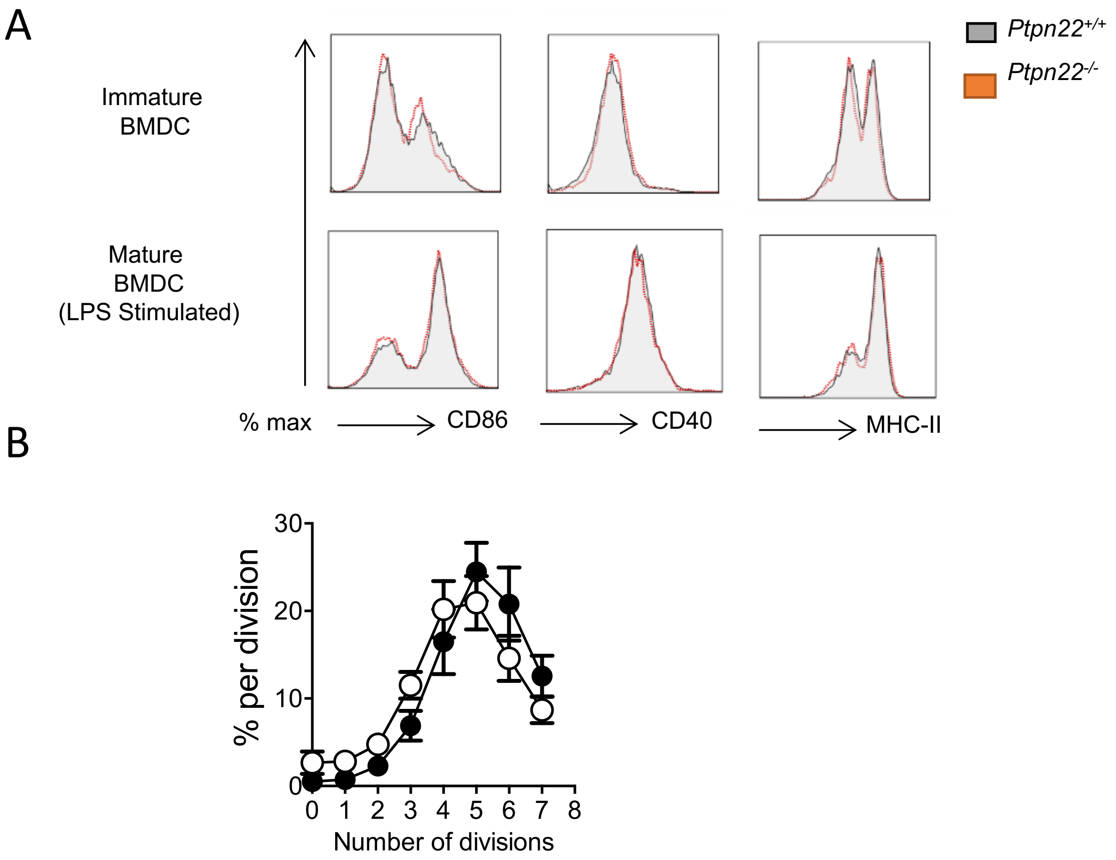

Supplementary figure S3. Ptpn22 is dispensable for LPS induced maturation of BMDC. (A) Day 6 WT and *Ptpn22*$^{-/-}$ BMDC were pulsed for 24 hours in the presence or absence of LPS. Cell surface expression of of maturation markers was determined by flow cytometry. Representative flow cytometry plot of CD86, CD40, MHCcII IA^{b}. Representative of 6-10 independent experiment. **(B)** CTV dilution of WT OT-II CD4^{+} T-cells co-cultured with WT (white) and *Ptpn22*$^{-/-}$ (black) LPS and OVA$^{323-339}$ pulsed BMDC for 6 days + 48 hour restimulation with fresh WT or *Ptpn22*$^{-/-}$ BMDC. N=6-7 per group; circles represent mean ± s.d.

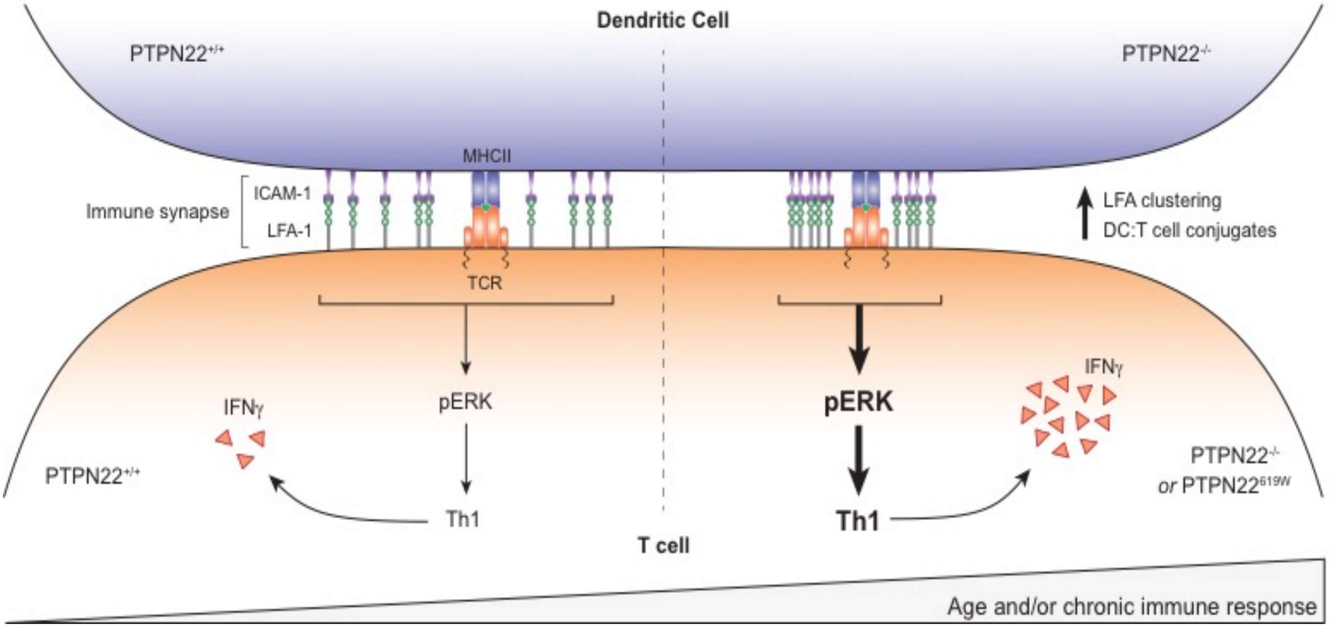

Supplementary Figure S4. Mechanistic model of Th1/IFNy regulation by PTPN22. In *Ptpn22*$^{-/-}$ and *Ptpn22*^{R619W} mice IFNy^{+} Th1 cells expand with age or following immune challenge. We propose that expansion of Th1 cells occurs via two processes; 1. PTPN22 negatively regulates LFA-1 induced Th1 cells by enhancing LFA-1 clustering, and immune synapse formation and conjugate formation and 2. repeated stimulation of T-cells with *Ptpn22*$^{-/-}$ BMDC enhances Th1 responses. Similar increases in LFA-1 clustering have been observed in human PTPN22^{620W} T-cells which manifests as increased Erk activation (Burn et al Sci. Signal. 2016), a key factor required for *Ifny* expression. We propose that overtime the PTPN22^{620W} risk-allele increases the propensity of T-cells to become IFNy^{+} altering the balance of effector T-cell and thereby increasing the risk of autoimmune disease development.

Species	T-cell responses	Associated findings	Reference
Human	Homozygote PTPN22^{620W} donor T cells show exaggerated IFNy and TNFα production; absence of suppression by Tregs.	Increased effector memory T cells, reduced Th17 responses. Increased pErk pAkt and pSLP-76.	Vang 2013 [1]
Human	PTPN22620W is associated with increased IFNy mRNA expression.	Findings reported in T-cells from Crohn's patients hetero/homozygous for C1858T.	Sharp 2018 [2]
Human	PTPN22^{620W} heterozygote carriers have increased circulating CD4+ memory T cells, but reduced TCR-induced IL-2 and IL-10 production. A non-significant trend towards increased IFNy expression is reported. T-cell proliferation is unaltered.	1858 T/T homozygotes associated with profound reductions in TCR signaling, including reduced TCR-induced calcium mobilisation, in genotyped healthy donors and type 1 diabetes patients.	Rieck 2007 JI [3]
Mouse	*Ptpn22* required for Treg induction. PTPN22 dispensable for IFNy.	Increased IFNy in the absence of PTPN22 observed under conditions of low anti-CD3 stimulation.	Fousteri 2014 [4]
Mouse	*Ptpn22* deficiency increases number and function of viral specific CD4+ T cells expression IFNy, TNF and IL-2. *Ptpn22*$^{-/-}$ are biased towards a SLAM^{hi} CXCR-5^{neg} Th1 phenotype.	*Ptpn22*$^{-/-}$ show increased CD4 dependent clearance of LCMV infection. Type I IFN responses and are impaired, while serum IL-10 levels are increased.	Maine 2016 [5]
Mouse	T-cell targeted overexpression of *Ptpn22* in transgenic NOD mice reduces IFNy expression and Th1 differentiation. High strength TCR stimulation can over-ride this effect.	Transgenic T cells demonstrated reduced TCR-mediated proliferation. Severity of insulitis and diabetes in transgenic NOD mice are attenuated. Transgenic Ptpn22 attenuates Erk phosphorylation.	Yeh 2013 JI [6]

Table 1. Regulation of Th1/IFNy responses by PTPN22 in man and mouse.

[1] T. Vang, J. Landskron, M.K. Viken, N. Oberprieler, K.M. Torgersen, T. Mustelin, K. Tasken, L. Tautz, R.C. Rickert, B.A. Lie, The autoimmune-predisposing variant of lymphoid tyrosine phosphatase favors T helper 1 responses, Hum. Immunol. 74 (2013) 574–585.

[2] R.C. Sharp, S.A. Beg, S.A. Naser, Role of *PTPN2/22* polymorphisms in pathophysiology of Crohn's disease, World J. Gastroenterol. 24 (2018) 657–670.

[3] M. Rieck, A. Arechiga, S. Onengut-Gumuscu, C. Greenbaum, P. Concannon, J.H. Buckner, Genetic variation in PTPN22 corresponds to altered function of T and B lymphocytes., J. Immunol. 179 (2007) 4704–10.

[4] G. Fousteri, T. Jofra, I. Debernardis, S.M. Stanford, A. Laurenzi, N. Bottini, M. Battaglia, The protein tyrosine phosphatase PTPN22 controls forkhead box protein 3 T regulatory cell induction but is dispensable for T helper type 1 cell polarization, Clin. Exp. Immunol. 178 (2014) 178–189.

[5] C.J. Maine, J.R. Teijaro, K. Marquardt, L.A. Sherman, PTPN22 contributes to exhaustion of T lymphocytes during chronic viral infection, Proc. Natl. Acad. Sci. 113 (2016) E7231–E7239.

[6] L.-T. Yeh, S.-C. Miaw, M.-H. Lin, F.-C. Chou, S.-J. Shieh, Y.-P. Chuang, S.-H. Lin, D.-M. Chang, H.-K. Sytwu, Different Modulation of Ptpn22 in Effector and Regulatory T Cells Leads to Attenuation of Autoimmune Diabetes in Transgenic Nonobese Diabetic Mice, J. Immunol. 191 (2013) 594–607.
